# Characterization of nano-silica vegetable grease with diffusing wave spectroscopy DWS and Raman spectroscopy

**DOI:** 10.1038/s41598-023-45669-0

**Published:** 2023-11-03

**Authors:** Jolanta Drabik, Rafal Kozdrach, Marian Szczerek

**Affiliations:** 1 Bioeconomy and Ecoinnovation Department, Lukasiewicz Research Network – Institute for Sustainable Technologies, 26-600 Radom, Poland; 2 Tribology Department, Lukasiewicz Research Network – Institute for Sustainable Technologies, 26-600 Radom, Poland

**Keywords:** Chemistry, Energy science and technology, Engineering, Materials science, Nanoscience and technology

## Abstract

The diffusing wave spectroscopy (DWS) method made it possible to identify changes in the dynamics of the free movement of particles in the grease under the influence of temperature, which changed the viscoelastic properties of the grease. Changes in the parameters determined by DWS method influenced changes in the chemical structure, which was confirmed by Raman spectroscopy, determining the integral intensity of the unsaturated to saturated bond bands found in the grease. The article presents the results of the influence of temperature on changes in the viscoelastic states of vegetable grease evaluated on the basis of properties determined by DWS (diffusing wave spectroscopy). The following parameters were used to evaluate the viscoelastic states: the intensity correlation function (ICF), the correlation function of mean square displacement (MSD), the elastic modulus G′ and the viscosity modulus G″. A significant effect of temperature on changes in the microstructure of vegetable grease was observed, which was reflected in the viscoelastic parameters. The dynamics of the free movement of molecules in the grease was changed, which affected the elasticity of the system and the displacement of the G′ and G″ modules towards higher frequencies.

## Introduction

In recent years, the use of vegetable oils as substitutes for petroleum-based raw materials has become widespread in lubrication technology. The expansion of their use as a component of industrial lubricants provides the basis for minimising the negative impact on the environment both during operation and after their performance has been depleted^1^.

The question of replacing classic industrial lubricants with ecological ones is still open due to the quantitative and qualitative constraints regarding the availability of biodegradable and non-toxic oil bases with increased oxidation resistance and functional additives, including being ash-free, and low in phosphorus and sulphur contents. This work focuses on the use of vegetable oils and synthetic products, which are an alternative to oils deriving from crude oil processing^2–5^. Currently, vegetable oils, epoxy natural oils, as well as inedible oils are used increasingly more frequently as bio-components of lubricants^6,7^.

An important limitation of the widespread use of natural oils in lubrication technology is their low oxidation resistance resulting from the presence of unsaturated bonds because these oils contain monounsaturated and polyunsaturated fatty acids. Differences in reactivity between oils depend on the number of double bonds, their mutual position and the geometric structure of the chain^8^.

The rate of oxidation of vegetable oils, however, is determined by the composition of fatty acids, the presence of pro-oxidants (chlorophylls, metals) and antioxidants (tocopherols, carotenoids, phenolic compounds, etc.), as well as storage conditions, with the most important role played by temperature, light and oxygen.

The solution to this problem can be both the chemical modification of the oils and the use of antioxidant additives, which cause the oxidation reaction to be interrupted at the initiation stage. The effectiveness of their action depends on the chemical structure of vegetable oils, while the selection of both type and quantity requires a number of experimental works^9^.

Lubricants, on non-toxic and biodegradable bases, are an alternative to commonly used engine, hydraulic, gear oils^10,11^. Despite clearly out lined trends related to improved environmental performance and numerous legal regulations, they still represent a small percentage of the global lubricants market. So far, most of the work on the assessment of the rheological properties of lubricants that are non-Newtonian liquids is based on the results obtained in tests of classical rotational rheology, with an increasing gradient of shear velocity or variable amplitude of deformations^7,12–14^. Lubricants belong to a widely used group, whose specific structural features primarily require the assessment of rheological and lubricating properties. DWS diffusion microrheology, as an analytical tool, is used to study non-Newtonian liquids in a wide frequency range. The literature review shows, that DWS diffusing wave spectroscopy is primarily used to study emulsions, suspensions, gels, and the colloidal systems of biopolymers in order to track sedimentation processes, structural changes during gelling or reversible processes of sol into gel.

The literature on the subject describes the results of research characterizing the rheological and structural properties of heterogeneous and homogeneous dispersion substances with a relatively low content of dispersed phase^15–17^. DWS is widely used to determine the dynamics of particle motion, changes in the structure and mechanical properties of emulsions, and to evaluate the physical stability of pharmaceutical emulsions based on the values of the MSD function over time, as well as changes in the values of the G′ and G′ modules from frequency, in order to explain the processes of emulsion destabilization^15–19^.

Using the DWS technique, studies were conducted that yielded information about the movement of particles in concentrated fluids, such as colloids and microemulsions^20^ and about the heterogeneity of biopolymers^21–23^. The development of the microstructure and mechanical properties of various types of gels was observed on the basis of changes in the value of the MSD correlation function^24–28^. The rheological characteristics and microstructure of submicron emulsions deposited on a modified maize-based wax starch matrix were determined, the change in the microstructure of the emulsions studied was evaluated, and the mechanism of structure destabilization was confirmed^29^. Using optical microrheology, the size of colloidal particles was measured, the gelling point was determined, and the process of destabilization of colloidal systems as well as the process of particle aggregation and monitoring of their displacements were described^30–32^. The rheological properties of micellar fluids and colloidal dispersions, polymers and biomaterials were determined on the basis of changes in MSD function values over time, and the relaxation modes of adsorbed polymers were evaluated^33,34^. Using the DWS technique, the rheological properties of high viscosity silicone oil were determined. The kinetics of its structure change was evaluated and a mathematical model describing its viscoelastic properties was proposed^32,35–39^. To sum up, the literature on DWS diffusion microrheology describes the results of research characterizing the rheological and structural properties of dispersive, heterogeneous and homogeneous substances with a diverse content of dispersed phase, such as emulsions, suspensions or polymers.

The analysis of the changes in the microstructure of dispersion systems, using DWS microrheology, as a result of the interaction between individual phases forming dispersion, although the subject of many studies, does not concern the assessment of the microstructure of greases. There is a gap in the literature regarding the assessment of the microstructure of greases at the molecular level in the linear viscoelasticity range using the DWS method. In this area, there are many unresolved issues regarding the identification of the complex rheological properties of lubricants affecting their functional characteristics. Therefore, DWS and Raman spectroscopy were used to analyze the transformations occurring in the microstructure of the developed vegetable grease under the influence of temperature. The DWS technique made it possible to measure the dynamics of local intermolecular displacements because it is based on the assumption that the transport of light in the media is associated with the diffusion of particles in the dispersing phase. Conversely, Raman spectroscopy, provided information on the chemical structure of the grease subjected to different temperatures during DWS measurements. Raman spectroscopy was chosen over spectral methods because it allows the monitoring of changes occurring at the molecular level. The results it yields are a continuation of the conducted research on the impact of modifications on changes in the degree of unsaturation of fatty acids present in the base vegetable oil^7^.

Raman spectroscopy can reveal many molecular aspects of a complex grease mixture by focusing on the intermolecular interactions of its self-organizing components. This information is particularly useful in the analysis of changes in the behavior of non-Newtonian systems such as lubricants, and in understanding aggregation and gelling in such systems. Raman spectroscopy is a widely used analytical technique to identify the chemical structure of, among others, polymer solutions, oil bases or lubricants treated with a heat medium^15–17^. The method is success fully used to assess changes in the chemical structure of colloidal systems^18^, paraffin waxes and polymer microemulsions^19^.

This paper presents the possibility of using the DWS method to evaluate the viscoelastic properties of a vegetable grease with a plastic consistency in its composition: dispersed phase in the form of an inorganic thickener and vegetable oil, as a dispersing phase, forming a colloidal system. In addition to evaluating the microstructure of the grease in the linear viscosity and elasticity ranges, the DWS method made it possible, to evaluate the influence of temperature on the reconstruction of the structure, as presented by the authors. An attempt was made to explain the changes occurring in the chemical structure of vegetable grease under the influence of temperature by means of Raman spectroscopy by determining the integral intensity of the bands of characteristic unsaturated to saturated bonds occurring in vegetable grease. Observation of the changes in the chemical structure of vegetable grease, based on Raman spectra, allowed for a comprehensive assessment of the effect of temperature on the transformation occurring in the microstructure of the tested lubricants at the molecular level, which affect viscoelastic properties.

## Experimental work

### Materials

On the basis of certified components, a vegetable lubricant was developed with beneficial antiwear properties and oxidative stability^40,41^. Vegetable oil from *Crambe Abyssinica* (Clariant) seeds^6,7^ was used as a dispersion phase for the preparation of the grease, while modified Aerosil® amorphous silica (Evonik Resource Efficiency) on particle sizes 7-40 nm was used as a dispersed phase^42,43^. From these selected components, a vegetable lubricant was produced, which was designated as Grease A. The lubricant composition was prepared by thorough homogenization of Aerosil® amorphous silica in Abyssinian oil. Mixing was performed at a spindle speed of 22,000 rpm for 30 minutes. Then tests were carried out, which showed that the prepared lubricant is a homogeneous substance. During particle size testing, it was confirmed that no agglomerates were formed, and the measured mean free path I* was similar in each case. Table 1 characterizes the properties of the base oil and grease A.

**Table 1:** Characteristics of *Crambe Abyssinica* base oil and vegetable nano-silica grease A.

Parameters	The method	Results
Basic oil A		
Kinematic viscosity, mm^2^/s	PN-EN ISO 3104:2021-03	
Temperature 40 °C		46.9 ± 1.7
Temperature 100 °C		10.1 ± 0.8
Viscosity index, VI	PN-ISO 2909:2009	208 ± 11
Viscosity class, ISO	PN-ISO 3448:2009	48 ± 3
Oxidation stability, PetroOxy test temperature 80 °C; oxidation induction time t _ON_, h	PetroOxy	32.8 ± 2.7
Antiwear properties, d, mm	PN-C-04147	0.59 ± 0.12
Grease A		
Penetration, mm/10	PN-ISO 2137:2021-07	267 ± 17
Consistency class	NLGI	NLGI 2
Dropping point, ^o^C	PN-ISO 2176:2011/A1:2021-07	244.5 ± 12.5
Oxidation stability, PetroOxy test temperature 80 °C; oxidation induction time t_ON_, h	PetroOxy	46.5 ± 2.8
Antiwear properties, d, mm	PN-C-04147	0.40 ± 0.17

An important factor affecting the destabilization of lubricants developed on the basis of vegetable oils is temperature. Therefore, the developed grease A was subjected to varying temperatures during DWS testing. The experiments were carried out at temperatures of 20 °C, 40 °C, 60 °C and 80 °C. Lubricants were designated as: Grease A_20, Grease A_40, Grease A_60 and Grease A_80.

### Methodology

#### Rheological tests

Tests on the rheological properties of vegetable grease after temperature changes were performed using DWS RheoLab Diffusing Wave Spectroscopy from Switzerland. The essence of DWS measurements consisted in monitoring the stochastic displacements of markers subject to position fluctuations in the tested grease. TiO_2_ nanoparticles were used as a marker.

During rheological studies on the DWS spectrometer, the size of TiO_2_ particles, which was used as a marker in rheological studies was examined. In each case, the TiO_2_ particle size was 360 nm±5, so there was no particle agglomeration process. And such an assumption was made that no aggregates are formed during the temperature increase, which was a key issue when interpreting the results of the MSD function.

Based on the DWS measurements, the following were determined: the correlation intensity function ICF (*intensity correlation function*), the correlation function of the mean square displacement of MSD (*mean square displacement*), the elastic modulus G′ and the viscosity modulus G″. In each of the rheological tests, the condition was met that the ratio of the thickness of the cuvette to the average free path was greater than 7 (L/I*> 7). Based on the analysis of the data, the elastic index EI was calculated and the change in the viscoelastic properties of the tested lubricants depending on the temperature was determined.

The ICF correlation intensity function characterizes the temporal fluctuations in the intensity of the scattered laser light after passing through the test sample^44–47^. The ICF function is described by the following formula:

1 $${g}^{\left(2\right)}\left(\tau \right)=\frac{\left[I\left(t\right)*I\left(t+\tau \right)\right]}{{\left[I\left(t\right)\right]}^{2}},$$

where I intensity of diffused light, t time, τ delay time.

A correlation function called the average displacement of the MSD molecule is determined by measuring the dynamics of the movement of the positions of the marker molecule in the fluid under study at specified intervals^48–52^. The MSD correlation function is specified by the following formula:

2 $$<\Delta r2\left(t\right)=<{[x\left(t+\theta \right)-x\left(t\right)]}^{2}+{[y\left(t+\theta \right)-y\left(t\right)]}^{2},$$

where x, y coordinates of the position of the marker molecule, t time, Θ time between consecutive positions of the marker.

The viscosity *G*′′(ω) modulus represents sinusoidal deformations proportional to the frequency^53–56^. This parameter is described by the formula:

3 $${G}^{{\prime}{\prime}}\left(\omega \right)={G}^{*}\left(\omega \right)\mathrm{sin}\left[\frac{\pi \alpha \left(\omega \right)}{2}\right],$$

where *G*^∗^ complex module, ω frequency, α(ω) logarithmic derivative of the MSD function after time.

The elastic modulus *G*′(ω) is a measure of elastic forces during cosine deformations proportional to frequency^53–56^. This parameter is described by the formula:

4 $${G}^{\prime}\left(\omega \right)={G}^{*}\left(\omega \right)\mathrm{cos}\left[\frac{\pi \alpha \left(\omega \right)}{2}\right],$$

where *G*^∗^ complex module, ω frequency, α(ω) logarithmic derivative of the MSD function after time.

The elasticity index EI is a measure of the elasticity of the tested sample^57–60^. This parameter is determined from the MSD function at the moment when the plateau characteristic for this function ends and the value of the correlation function begins to increase. This parameter is determined from the MSD function. The indicator was calculated according to formula (5), and the obtained parameters are presented in Table 2:

**Table 2:** Change of elasticity index and G′ and G″ modules in various temperatures for the tested vegetable grease A.

Tested greases	DWS parameters		
	Elasticity index EI μm^–2^	Values of modules in crosscut point G′=G″, Pa	
		In first crosscut point	In second crosscut point
A_20	2.51·10^6^ ± 0.12·10^6^	112 ± 17	835 ± 52
A_40	2.14·10^6^ ± 0.17·10^6^	148 ± 11	1892 ± 211
A_60	1.81·10^6^ ± 0.22·10^6^	272 ± 27	2842 ± 244
A_80	2.04·10^6^ ± 0.23·10^6^	462 ± 34	2908 ± 294

5 $$EI=1/<\Delta r2(\tau )>max.$$

The slope of the MSD function determines the nature of the sample to be tested. This parameter is determined from the formula^61–64^:

6 $$\Delta {r}^{2}\left(\tau \right)>{\tau }^{n},$$

where τ time, n slope of the MSD function.

The inclination value of the MSD function is in the range of 0-1 for viscoelastic fluids, (1 for Newtonian fluids, and 0 for a perfectly elastic body).

The D_0_ and D_m_ diffusion coefficients characterizing the behavior of suspended particles in liquids under the influence of variable temperatures and concentrations at short and long delay times of the correlation function MSD were determined from the Belloure equation^53^:

7 $$<\Delta {r}^{2}\left(\tau \right)=6{\delta }^{2}\left(1-{exp}^{-\left(\frac{{D}_{0}}{{\delta }^{2}}*t\right)}\alpha \right)-1/\alpha {(1+\frac{{D}_{m}}{{\delta }^{2}}*\tau )}^{-\beta },$$

where τ lag time, δ amplitude of particle movement, α parameter characterizing a wide spectrum of relaxation times in a plateau with short delay times for MSD functions, D_0_ diffusion coefficient at short delay times of MSD function, D_m_ diffusion coefficient at long delay times of MSD function, β parameter characterizing changes in the slope of the MSD correlation function with long delay times describing the diffusion area of the tested sample.

The number of scattering events is *N* = *s*⁄*l*, where *l* is the mean path length between the scattering events and s is the photon path. The average distance for a photon to see its propagation direction randomized, called the transport mean free path I*,which carried out automatically by the optical rheometer according with the formula:

8 $${I}^{*}=\frac{l}{(1-(cos\theta )},$$

where 〈cos θ〉 is the mean of the cosinus of the angle $\theta$ the photon is scattered by the particles within the photon transport process. Small particles (size much smaller than the wavelength, i.e. Rayleigh-Gans-Debye limit) scatter isotropically and 〈cos(ϕ)〉 ≈ 0, hence *l*∗ ≈ *l*. However, larger particles scatter preferentially in forward direction, and thus *l*∗ ≫ *l*.

While the distribution of photon paths *P*(*s* = *Nl*), is appointed automatically from apparatus with the formula:

9 $${g}_{1}\left(\tau \right)=\underset{0}{\overset{\infty }{\int }}{d}_{s}P\left(s\right){e}^{{1}/{6\langle q2\rangle \langle \Delta r2\left(\tau \right)\rangle N}}.$$

The key assumptions were made in the experimental studies, namely: I* was determined by the concentration and size of particles, laser properties and properties of the suspending medium; particles in the lubricant (grease is not a suspension but a dispersion system) are monodisperse, which has been proven by particle size testing, while P(s) has been determined from the distribution of photon path lengths in the sample geometry (size determined automatically by the apparatus based on available formulas in accordance with the theory of diffusion spectroscopy.

In order to perform the correct tests, the necessary experiments were carried out regarding the calibration of the device, the selection of the measurement mode and the thickness of the cuvette. Calibration of the device was carried out using a polystyrene standard with a precisely defined particle size (222 nm). The selection of the cuvette for the tested media is very important, because correct measurements require the condition that the ratio of the cuvette thickness (L) to the mean free path of the particle (l*) must be greater than 7 and less than 30. Measurements were made in the backscattering mode. (backscattering) in order to determine the best optical path and obtain correct results of the ICF function of light propagation in the diffusion process.

The advantage of the tests carried out using the DWS technique is the possibility of recording changes in the dynamics of particle motion under the influence of thermal excitation, which in this case guarantees obtaining changes in viscoelastic states in the dispersion caused by microscopic changes.

The principle of operation of the DWS rheometer is based on the assumption that light transport can be treated as a diffusion process in optically turbid samples. The apparatus enables microrheological measurements of materials in the conditions of static intermolecular displacements in a wide range of frequencies and viscoelasticity. The optical rheometer enables testing in two different geometries, i.e. transmission and backscatter [EU patent 1720000]. In transmission mode, scattered light is detected as it passes through the sample, and variations in intensity are correlated using the Correlation Intensity Function (ICF). In contrast, in the backscatter mode, the light that is scattered back towards the incident beam is collected and its fluctuations are measured.

Rheological measurements were carried out at 20 °C in three repetitions. Based on the analysis of the determined rheological parameters, the change in viscoelastic properties of the initial samples of lubricants and after thermal excitation was determined. Before starting the measurements, the apparatus was calibrated using a standard, which is a polystyrene emulsion with a particle size of 222 nm in water. Then, the refractive index for the oil base was determined, the duration of the measurement and the size of the spectrometric cuvette in which the tested samples would be placed were set, which were prepared by adding a marker to their structure, which is titanium dioxide with a particle size of 360 nm and homogenizing by mixing the resulting grease for 20 minutes at a spindle speed of 15,000 rpm. Rheological tests were carried out in a measuring cuvette with an optical path of 1 mm. The duration of the measurement was 90 s. For the statistical evaluation of the results, the Q-Dixson test was used with a confidence level of 95%.

#### Spectral tests

Raman spectra were obtained using the confocal mesh microspectrometer Raman NRS 5100 (Jasco Corporation, Japan) equipped with an excitation laser with a wavelength of 532 nm and a CCD detector. The working conditions of the spectrometer were as follows: diffraction grid 1800 lines/mm, laser power 5.1 mW, numerical aperture 4000 μm, resolution 8.4 cm^–1^, lens magnification 20x, exposure time 15 s. Surface areas in the range of characteristic bands found in vegetable oil were calculated using the mathematical apparatus available in *the Jasco V500/FP-750 program*.

### Statement regarding plant guidelines

The use of plant parts in the study complies with international, national, and/or institutional guidelines.

### The source origin of Abyssinian oil from *Crambe Abyssinica* seeds

The *Crambe* *Abyssinica* seed oil was purchased in France.

## Results and discussion

The obtained rheological parameters of lubricants in the range of linear viscoelasticity were, expressed by: function of ICF correlation intensity, correlation function of the mean square displacement of MSD, elasticity modulus G′ and viscosity modulus G″. These parameters are individual echoes characterizing the microstructure of the grease under the test. The mean square displacement of the MSD as a function of the decorrelation time is an individual identifier, i.e. the viscoelastic finger print of the grease under test.

**Figure 1:**
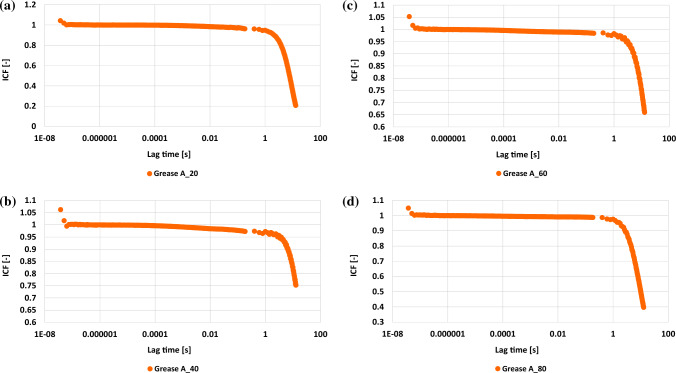
Dependence of the ICF intensity correlation function on time. Vegetable grease after tests at: (**a**) 20 °C, (**b**) 40 °C, (**c**) 60 °C and (**d**) 80 °C.

**Figure 2:**
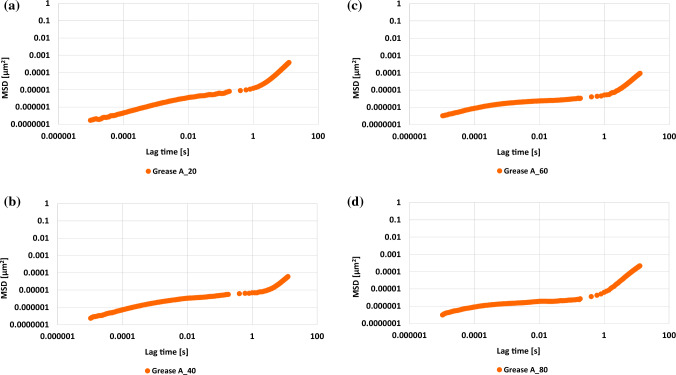
The dependence of correlation function MSD on time at a temperature of: (**a**) 20 °C, (**b**) 40 °C, (**c**) 60 °C and (**d**) 80 °C.

**Figure 3:**
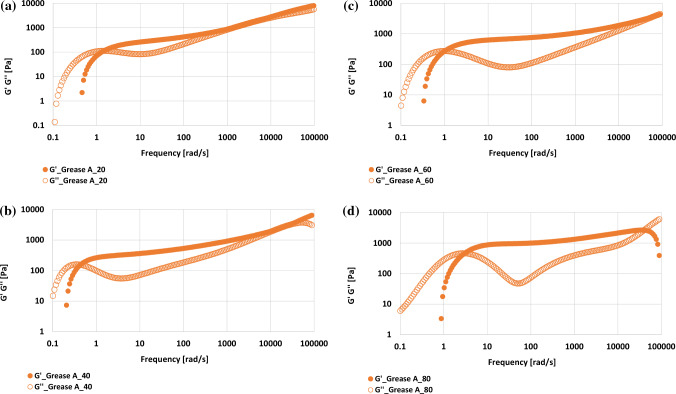
The dependence of storage modulus G′ and loss modulus G′’ on frequency at temperatures of: (**a**) 20 °C, (**b**) 40 °C, (**c**) 60 °C and (**d**) 80 °C.

For the prepared grease lubricant, the rheological properties were determined in the following temperatures: 20 °C, 40 °C, 60 °C and 80 °C. Then the influence of the applied temperature on the changes in rheological parameters was evaluated, (Table 2). The obtained test results are shown in Figs. 1, 2 and 3.

Figure 1 presents the dependence of the ICF (intensity correlation function) on the lag time for the vegetable oil lubricant at the four different temperatures at which measurements were carried out. The decay of the ICF function determined for grease A depends on the temperature and is slower at higher temperatures, suggesting slower dynamics of scattering particles. At temperatures of 40 °C, 60 °C and 80 °C, slower dynamics were observed directly related to the increased viscosity of the dispersion phase. ICF curves are converted to mean square displacement (MSD) curves.

The ICF decorrelation curve is a reflection of the speed of movement of particles and make it possible to calculate the average mean square displacement (MSD), the value of which depends on the distance travelled by the particles at a given time of decorrelation. Figure 2 shows the dependence of the mean square displacement function (MSD) on the lag time for the tested lubricating grease at four different temperatures in which the tests were carried out.

Analyzing the shape of the MSD correlation function, three phase scan be distinguished. In each of the discussed phases, a parameter was determined on the basis of which the microstructure of the tested lubricating grease can be characterized.

In the first phase, with short lag times, the value of this function increases. In the second phase, stabilization follows and the correlation function values do not change significantly. In this phase, the modulus of elasticity was determined. Called the plateau, this phase was characterized by the absence of changes in the value of the MSD function. It further, defines the size of elastic properties and characterizes the microstructure stability of the tested grease. In this phase, the value of the elasticity coefficient for the initial grease and after heating at higher temperatures was determined. For the initial grease A_20 (20 °C), this coefficient was lower than for grease after testing at higher temperatures. The value of the discussed parameter increased, which testifies to the weaker elastic properties of the grease at the higher temperatures in relation to the initial sample at 20 °C and to the change in the microstructure of the tested grease.

In the third phase, the value of the correlation function increases again. In this phase the diffusion coefficient at high values of lag time of the discussed function, as well as the slope coefficient of the MSD correlation function were designated. This value of the discussed parameter testifies to the high dynamics of the movement of the particles of the tested grease, and to a significant change in the speed of movement of the particles of the discussed samples. The value of the slope coefficient of the MSD correlation function for grease subjected to higher temperatures decreased, which could be caused by an increase in the distance between particles, a decrease in the dynamics of particle movement, and, thus a change in the microstructure of the tested grease subjected to a higher temperature.

It has been observed, that temperature changes the course of the MSD function of the tested vegetable grease. This indicates a change in the viscoelasticity of the grease. It was found that the higher the temperature acting on the grease, the lower values of the plateau of the MSD function. At a temperature of 80 °C, the MSD function was formed in a lubricant of microstructures and was characterized by higher elasticity than at 20 °C.

The viscous properties of the tested grease were analyzed on the basis of the slope of the curve of the MSD correlation function, with long lag times. In the adhesion of the inclination of the correlation function, MSD indicates an increase in the dynamics of the movement of molecules, and thus the advantage of the viscous module, which also indicates a change in the microstructure of the tested grease. It was found that the linear increase in MSD function occurs faster when DWS tests are performed at elevated temperatures. It was observed that at temperatures of 40 °C (A_40 grease), 60 °C (A_60 grease) and 80 °C (A_80 grease), there is a previously linear increase in the MSD function, denotes the higher viscosity of these lubricants than at 20 °C. On the basis of the results obtained by the DWS technique, the influence of temperature on the rheological characteristics of vegetable grease A was evaluated, which made it possible to classify the tested vegetable lubricant in terms of elasticity and viscosity:

$$ {\text{Elasticity}}:{\text{ grease A}}\_{8}0 > {\text{grease A}}\_{6}0 > {\text{grease A}}\_{4}0 \, > {\text{grease A}}\_{2}0, $$

$$ {\text{Viscosity}}:{\text{ grease A}}\_{4}0 > {\text{grease A}}\_{6}0 > {\text{grease A}}\_{8}0 > {\text{grease A}}\_{ 2}0. $$

Temperature changed the dynamics of the system, resulting in a change in the MSD function and the modulus of elasticity G′ and viscosity G″. The full characteristics of grease A after temperature changes are presented in the form of viscoelastic spectra (Figure 3).

Figure 3 shows the dependence of elasticity modulus G′ and viscosity modulus G″ on the frequency for the tested lubricating grease at four different temperatures in which the tests were carried out. The changes occurring in the linear viscoelasticity range of the tested lubricants depending on the temperature are reflected in the following modules: elastic G′ and viscosity G″. It was observed that the action of temperature influenced changes in the position of the intersection point of modules G′ and G″, (Fig.3). A temperature of 40 °C and 60 °C caused the G′ and G″ curves of A_40 and A_60 grease to move towards lower frequencies compared to A_20 grease. In the case of A_80 grease subjected to a temperature of 80 °C, it was observed that the position of the "G′ and G″ curves shifts towards higher frequencies compared to A_20 grease. It was found, that the increased temperature affects changes in the viscoelastic properties of grease A, which indicates the reconstruction of the microstructure of vegetable grease A subjected to the temperature. It was observed, that temperature influenced the dynamics of particle movements and the formation of a new microstructure in grease A, which resulted in a change in the value of the elastic index and a displacement in the curves of the G′, and G″ modules towards different frequencies than the position obtained for the grease at a temperature of 20 °C, (Tables 2, 3).

**Table 3:** Change of amplitude of motions particle, slope of MSD and diffusion indexes in various temperatures for the tested vegetable grease A.

Tested greases	DWS parameters			
	Amplitude of motions of particle 6δ^2^ , plateau, µm^2^	Slope of MSD, n, –	Diffusion index	
			In short time, D_0_ m^2^/s	In long time, D_m_ m^2^/s
A_20	1.66·10^-6^± 0.08·10^-6^	0.93± 0.03	5.56·10^-2^± 0.33·10^-2^	7.23·10^-5^± 0.41·10^-5^
A_40	1.06·10^-6^± 0.11·10^-6^	0.96± 0.02	3.72·10^-2^± 0.35·10^-2^	6.46·10^-5^± 0.44·10^-5^
A_60	1.00·10^-6^± 0.13·10^-6^	0.98± 0.02	3.14·10^-2^± 0.37·10^-2^	5.76·10^-5^± 0.47·10^-5^
A_80	0.61·10^-6^± 0.17·10^-6^	0.91± 0.03	3.66·10^-2^± 0.38·10^-2^	6.06·10^-5^± 0.52·10^-5^

In order to determine the quantitative differences in the microrheological behavior of grease A after temperature action, based on MSD curves, the elastic index EI was calculated according to formula (5), alongside the coefficient of inclination of the MSD curve at short and long decorrelation times. Based on the dependence of modules G′ and G″ on frequency, the values of the modules in the first and second cross cut points G′=G″ were determined. The results are given in Tables 2 and 3.

The values of the short-term and long-term diffusion coefficients indicate the sub-diffusion nature of the tested samples and the stability of the lubricant. A decrease in the values of the D_m_ and 6δ^2^ coefficients indicates an increase in the stability of the lubricant. The lower values of the D_m_ coefficient in relation to the D_o_ coefficient prove that the relaxation process of the tested samples is slower, which is related to the temperature increase. The viscosity value of the base oil is more important in the short lag times of the MSD function than the longer ones, which proves the greater ability of the tested samples to increase their stability. With increasing temperature, the crosscut point of G′ and G″ modules reaches higher values, both at the first and second points. The obtained results confirm the changes in the grease microstructure under the influence of temperature.

The presented results indicate that DWS is a very sensitive technique, which makes it possible to identify changes at the molecular level. The conducted research confirmed, that on the basis of measurements of particle movement in the dispersion system made using the DWS technique, consisting in determining the correlation function of the mean square displacement of MSD, the stability of the grease microstructure can be evaluated. The rheological parameters determined by the DWS method are an individual feature characterizing the microstructure of the lubricant and can be used to identify factors causing destabilization of the microstructure.

**Figure 4:**
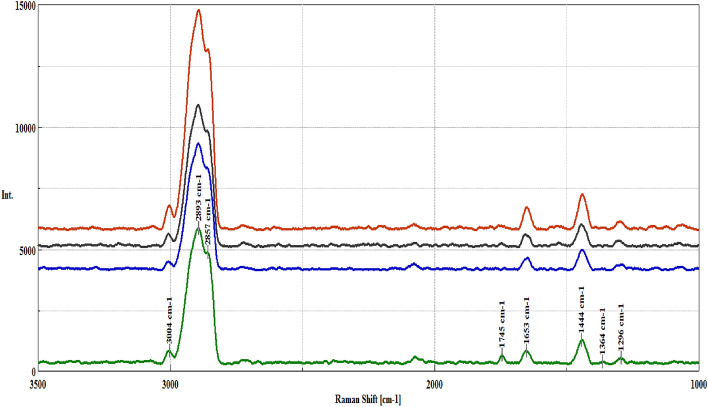
The Raman spectra of vegetable grease at temperatures of: 20 °C, 40 °C, 60 °C and 80 °C; A_20 at 20 °C (green line), A_40 at 40 °C (grey line), A_60 at 60 °C (blue line), A_80 at 80 °C (red line). (For read ability purposes, the spectra have been shifted).

Samples after temperature exposure in the DWS tests were analyzed using Raman spectroscopy to evaluate changes in structures from vegetable grease. The analysis of Raman spectra of vegetable grease A made it possible to study changes occurring under the influence of temperature in the range of characteristic bands of multiple unsaturated bonds and single saturated bonds occurring in fatty acids of vegetable base oil. Figure 4 shows the Raman spectra obtained for grease A at various temperatures (20 °C, 40 °C, 60 °C and 80 °C) in DWS tests. One way to obtain information about changes occurring at the molecular level in vegetable grease is to monitor changes based on the characteristic bands present in fatty acids of vegetable oil, such as:

1. The excited bands at 1266 and 1301cm^–1^, which correspond to the tensile and bending modes of unsaturated and saturated bonds between oil molecules;
2. Bands excited by tensile vibrations of groups C=C, scissor vibrations CH_2_ near 1654 and 1440cm^–1^ respectively;
3. Bands excited by tensile vibrations = C–H and C–H of groups CH_3_ and CH_2_ occurring near 3003 and 2859cm^–1^.

For the analysis of changes occurring under the influence of temperature in the structure of grease A, bands excited by tensile vibrations of unsaturated groups C=C located at 1654cm^–1^ and saturated groups CH_2_ occurring at 1440cm^–1^ were selected, because these bands are definitely separated and their boundaries are clearly marked. The integral intensity of the bands was determined as the value of the integral corresponding to the area under the peak. The ratio of the number of double bonds C=C to single C–C found in fatty acids of vegetable grease was determined, and, thus, the effect of increased temperature on changes in the structure of the grease was estimated by evaluating the degree of insatiability. The integral intensity of the I_1654_/I_1444_ bands resulting from the vibrations of the characteristic unsaturated bonds found in the vegetable grease was calculated, indicating the degree of its insatiability.

**Figure 5:**
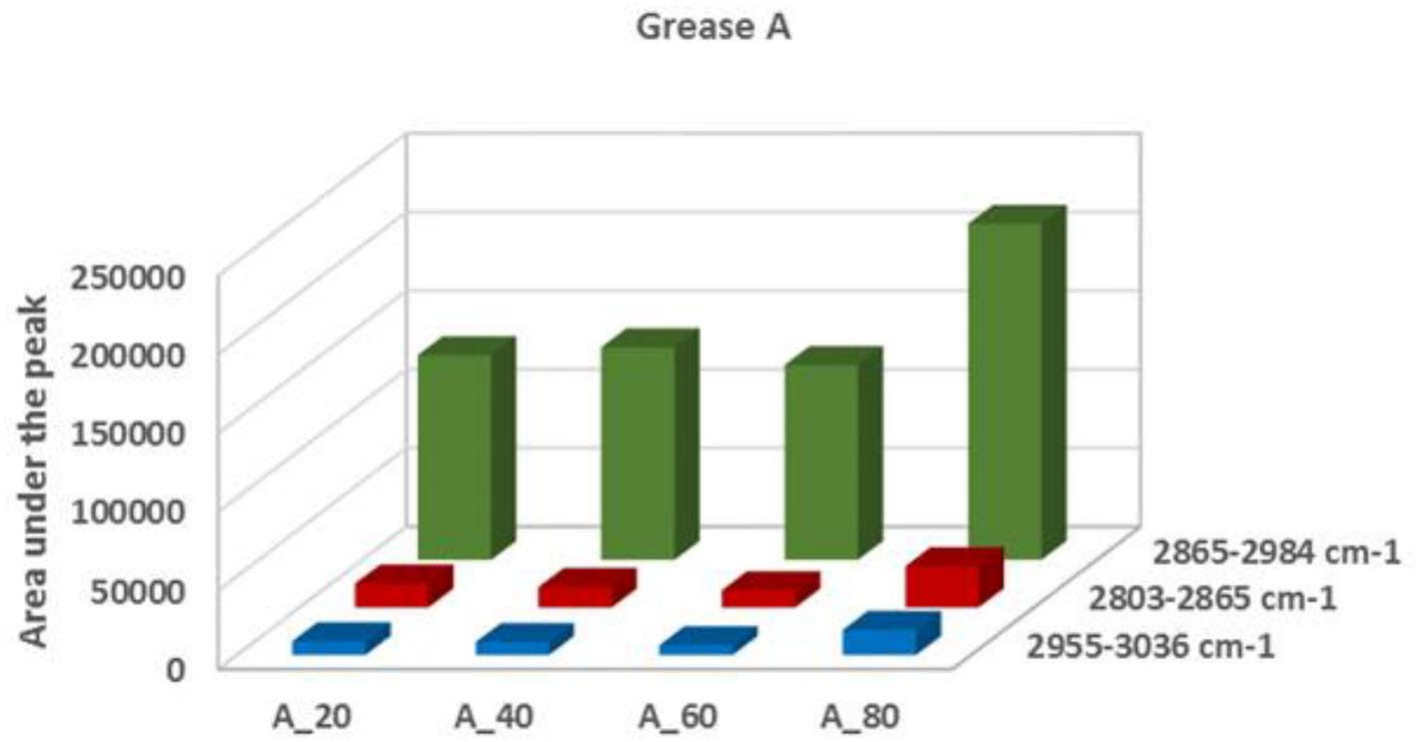
The influence of temperature on changes in the area under peak of Raman band in terms of the function group of fatty acids from vegetable base oil in grease A.

In the spectrum of grease A_20, a band resulting from the stretching vibrations of the ester bond (C=O) located at 1745cm^–1^ was observed. This band is connected with the CH_2_ groups (2850–2885cm^–1^). It was found, that during the action of increased temperature on vegetable grease, the band at 1745cm^–1^ disappeared, and the bands associated with the CH_2_ and CH_3_ groups were more intense. In the spectrum of the tested lubricants A_40, A_60 and A_80 an increase in the intensity of the bands characteristic of the functional groups of polyunsaturated fatty acids was observed in the ranges: 2985–3036 cm^–1^, 2865–2984 cm^–1^, and, 2803–2865 cm^–1^, which proves the changes occurring in the grease structure as a result of the action of increased temperature. It was assumed, that the measure of the changes occurring in the structure of the base oil is the surface area "under the peak", i.e. the area limited by the Raman intensity curve and the baseline in the range of wave numbers appropriate for the areas adopted as characteristic of the transformations occurring in the base vegetable oil. For the characteristic signals indicating the progress of changes, the surface areas of the bands accepted for analysis were calculated, (Fig. 5). It was found, that for A_80 vegetable grease, after exposure to a temperature of 80 °C in the DWS tests, the bands of the CH_2_ and CH_3_ groups were significantly larger than in the case of other lubricants (A_20, A_40 and A_60).

On the basis of the analysis of Raman spectra obtained for vegetable grease A, the influence of temperature on changes in the microstructure of vegetable grease was evaluated (Tables 4, 5).

**Table 4:** Influence of temperature on changes on the integral intensity of band I_1654_/I_1440_.

Grease	Raman spectroscopy integral intensity of bands	
	I_1654_/I_1444_	% Change after high temp
A_20	0.3943 ± 0.07	–
A_40	0.4722 ± 0.05	+ 19.75 ± 1.7
A_60	0.4637 ± 0.05	+17.60 ± 1.4
A_80	0.5151 ± 0.04	+30.64 ± 1.8

**Table 5:** Influence of temperature on changes on the elastic index EI.

Grease	DWS parameters	
	Elasticity index EI [m^2^]	% Change after high temp
A_20	2.51·10^6^± 0.21 ·10^6^	–
A_40	2.14·10^6^± 0.17 ·10^6^	− 14.74± 1.3
A_60	1.81·10^6^± 0.19 ·10^6^	− 27.89± 1.7
A_80	2.04·10^6^± 0.12 ·10^6^	− 18.73± 1.9

The use of Raman spectroscopy made it possible to evaluate the influence of temperature on changes in the microstructure of vegetable grease. It was observed, that the temperature increased the integral intensity of the analyzed bands I_1654_/I_1444_. For grease A_40 and A_60 subjected to lower temperatures, the change in integral intensity of the bands was 19.75% and 17.6%, respectively, compared to A_20 grease. A significant change, at a level of 30.64%, occurred at a temperature of 80 °C compared to grease A_20. This indicates significant changes in the structure of the grease under the influence of temperature. The degree of unsaturation of fatty acids determined on the basis of the intensity ratio I_1654_/I_1444_ increased compared to grease A_20. For vegetable grease A, after exposure to higher temperatures, each of the DWS tests showed an increase in the degree of unsaturation of fatty acids. This increase was the largest for A_80 grease.

**Figure 6:**
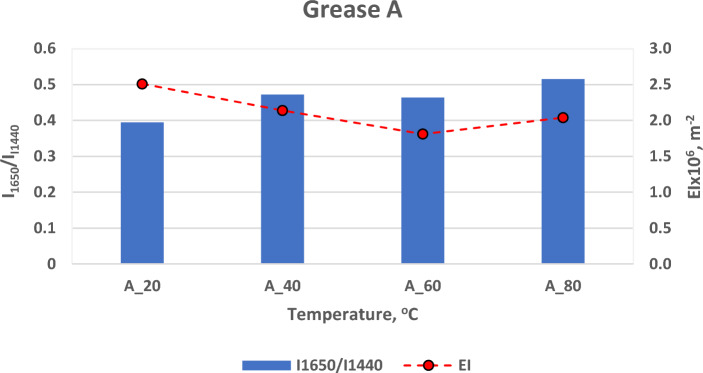
Influence of temperature on changes in the integral intensity of band I_1654_/I_1440_ and the elasticity index EI.

The identified bands present in the Raman spectrum of the tested vegetable grease I_1656_/I_1444_ characterizing the degree of unsaturation of fatty acids were compared with the elastic index EI, the viscoelastic parameter determined by the DWS method (Fig. 6).

The action of elevated temperature in DWS studies led to a change in viscoelastic parameters, including the value of the EI elastic index of the tested vegetable grease and the microstructure of vegetable grease. Raman spectroscopy was successfully used to identify changes in the structure of vegetable lubricants.

## Conclusion

The tests carried out confirmed, that the DWS technique can be used to evaluate the viscoelastic properties of lubricating greases. Most of the work on the measurements of the viscoelastic properties of lubricants has been based mainly on research conducted through oscillatory tests with an increasing gradient of shear velocity. So far, the DWS method, which allows for the non-invasive assessment of their microstructure, has not been used to analyze viscoelastic states. The proposed measurement method can, therefore, be valuable in the case of evaluating the viscoelastic properties of complex formulations without interfering with their structure, which allows for a better understanding of their cross-linking and microstructure. The analysis of the particle displacement phenomena at the microscale gives the opportunity to obtain the viscoelastic parameters in higher frequency ranges, unattainable for macroscopic studies in classical rheometry.

Thanks to the use of DWS, added knowledge was obtained about changes in viscoelastic states occurring in the microstructure of grease under the influence of temperature. The analysis of Raman spectra after DWS tests made it possible to evaluate the influence of temperature on changes in the structure of vegetable lubricants, in the range of unsaturated bands, indicating a change in the degree of insatiability. Innovative studies on the viscoelastic properties of lubricants at the molecular level using DWS and Raman spectroscopy allowed to monitor changes in the microstructure of vegetable grease under the influence of temperature. The experiments carried out confirmed the significant influence of temperature on the constitution of new stable microstructures for vegetable lubricants, which was reflected in their chemical structure.

## Data Availability

The data that support the findings in the present study are available from the corresponding author upon request.
